# Graph-Theory Algorithm for Prediction of Electrolyte Degradation Reactions in Lithium- and Sodium-Ion Batteries

**DOI:** 10.3390/ma18040832

**Published:** 2025-02-14

**Authors:** Lyuben Borislavov, Alia Tadjer, Radostina Stoyanova

**Affiliations:** 1 Institute of General and Inorganic Chemistry, Bulgarian Academy of Sciences, 1113 Sofia, Bulgaria; radstoy@svr.igic.bas.bg; 2 Faculty of Chemistry and Pharmacy, University of Sofia, 1164 Sofia, Bulgaria

**Keywords:** lithium-ion batteries, electrolytes, parasitic redox reactions, graph theory, cheminformatics, DFT

## Abstract

The growing demand for sustainable energy storage devices requires the fabrication of novel materials for rechargeable metal-ion batteries. The stability of the materials incorporated in the electrochemical cells plays a crucial role in the specific capacity and cycling stability of energy storage devices. The processes that occur inside such systems are fairly complex; hence, the identification of unwanted side reactions affecting the electrochemical stability is not a trivial task. The present study combines cheminformatics and quantum chemistry approaches to create an algorithm that generates diverse viable side products of redox reactions that a given electrochemical system, e.g., different cathode or anode materials, electrolytes, solvents, etc., can undergo. Two case studies of electrolyte degradation are presented: namely, ethylene carbonate (EC) and diglyme (DG). The effect of the electrode surface is modeled by the dehydrogenation reactions of the electrolyte solvents. The predicted degradation products after reduction and oxidation are validated using previously reported experimental data. For EC, the predicted products are CO, CO_2_, ethene, ethylene oxide, [CO_2_]^•−^, and [CO_2_]^•+^, while for DG alkoxy anions are mainly anticipated. The number of gaseous products formed upon DG degradation is significantly smaller than the number of gaseous species formed by EC fragmentation. The proposed algorithm opens new avenues for the rapid deduction of degradation products of novel electrolyte solvents for which no experimental data are available and can easily be adapted to predict the degradation of other materials.

## 1. Introduction

Lithium- and sodium-ion batteries (LiIBs and NaIBs) are emerging as the most promising energy storage technologies, combining high energy and power performance with affordability [1,2]. Both LiIBs and NaIBs operate using the same mechanism, which involves the reversible transfer of alkali ions between two electrodes via an electrolyte, followed by the redox interaction (simultaneous addition/removal of electrons) of Li^+^/Na^+^ with the electrodes [3]. The common operating mechanism of LiIBs and NaIBs underlies several degradation issues at different levels, i.e., battery, cellular, and individual levels [4,5]. At the battery level, there is a need for a detailed understanding of battery performance from the beginning to the end of the battery life [6]. At the cellular level, the important parameters responsible for cell degradation are the environment temperature, the state-of-charge, and depth-of-discharge, as well as the temperature variation within the cell [7,8]. At the individual level, degradation processes refer to those that cause the capacity to fade during cycling due to the electrochemical instability of the electrolyte, anodic metal plating, solid electrolyte interphase growth, and loss of contact between electrode particles [9]. Given the degradation processes complexity, recent research has focused on selecting methods that not only detect but also predict degradation or mechanical stress products [10].

In most practical cases, the electrodes of LIBs contain layered/spinel oxides, polyanionic compounds, alloys, or carbonaceous materials, while the electrolyte consists of a lithium salt dissolved in a carbonate-based solvent [3]. In this cell configuration, the main electrochemical reaction is usually concomitant with parasitic side reactions, including surface electrode–-electrolyte interactions, as well as electrochemical degradation of the electrolyte, which in turn is responsible for shortening the battery life [11]. To overcome this drawback, research in recent years is dedicated mainly to understanding degradation reactions at the individual level and their correlation with cell performance [6]. In this context, a variety of experimental techniques (both in situ and ex situ) and quantum chemical methods have been used, making some parasitic side reactions involving electrode materials somewhat easier to predict and experimentally authenticate [12,13]. In contrast, electrolyte degradation reactions are less foreseeable due to the formation of multiple different products and the difficulty in detecting and identifying intermediates [14,15]. In general, experimental and quantum chemical methods are time-consuming. This requires the development of new efficient approaches to analyze electrolyte degradation reactions.

Machine learning methods have been successfully utilized to model different components of lithium-ion cells, despite the fairly low number of openly available battery datasets [16]. Classical cheminformatics approaches and machine learning, on the other hand, have proven their ability to determine possible products of chemical reactions. These kind of methods have successfully been applied in synthesis planning [17], retrosynthetic analysis [18], mass-spectroscopy fragmentation forecasting [19] and the prediction of redox activity in quinones-based electrodes [20]. Machine learning methods allow quick calculations to be made about the state of the battery, but fail to understand the physical mechanisms involved [21]. Due to the lack of datasets on lithium-ion cell degradation products [22], it is hard to apply supervised machine learning techniques for cell degradation products prognosis.

To address this issue, we developed a graph-theory top-down approach that generates diverse feasible redox reactions a given electrochemical system can undergo. Two case studies on electrolyte components degradation are presented, namely ethylene carbonate (EC) and diglyme (DG). These electrolyte solvents are selected because they are among the most widely used and studied solvents in electrolytes for LiIBs and NaIBs [23,24]. To account for the electrode surface, dehydrogenation and subsequent degradation of the electrolyte solvents is provisioned, motivated by existing experimental and theoretical findings for dehydrogenation of EC on oxide electrodes surfaces [25,26].

## 2. Methodology

### 2.1. Basics of Graph Theory

Since its formulation by Euler in 1736 [27] the graph theory has found numerous applications in many different realms of science, technology, and even in economics and sociology [28]. Graph theory proved itself to be particularly useful in chemistry, since the graph representation is capable of providing machine-readable description of molecules capturing intrinsic properties of their chemical structures [29]. Formally, a graph G(V, E) can be defined as a nonempty set of vertices V = {*V_i_*} and by a set of edges E = {(*V_i_*, *V_j_*)} joining different pairs of vertices. If the tuples (*V_i_*, *V_j_*) are unordered, the graph is referred to as undirected, while for ordered tuples the graph is designated as directed. The degree of a vertex *V_i_* in a graph is defined as the number of edges containing *V_i_*. Two vertices are said to be adjacent if they are connected by an edge, while two edges are adjacent if they have a common vertex. For a graph with N vertices, it is possible to define an (N × N) symmetric square adjacency matrix **A** = [*a_ij_*] as follows: $a_{ij}=1\;if\;\left(V_{i},\;V_{j}\right)\;\in E$ (i.e., vertices *V_i_* and *V_j_* are connected) $a_{ij}=0\;if\;\left(V_{i},V_{j}\right)\notin E$ (i.e., vertices *V_i_* and *V_j_* are not connected).

### 2.2. Chemical Graph Theory

A central concept in the chemical graph theory is the so-called constitutional graph, in which atoms are represented as vertices and bonds are represented as edges. For denoting double bonds and lone electron pairs, so-called pseudographs are introduced. The pseudographs may contain loops (loop is an edge connecting a vertex to itself) indicating lone pairs and multiple edges signifying bonds of higher order. In both constitutional graphs and pseudographs, the hydrogens can be omitted (the number of hydrogens connected to an atom are calculated by the atom valence) [30]. Chemical graph theory has been extensively used in the fields of quantitative structure–properties relationships (QSAR), chemical fingerprints generation, retrosynthetic analysis, and mass-spectroscopy fragmentation prediction [31], which prompted the assumption that it can be employed for prognosis of parasitic side-reaction products in metal-ion batteries as well.

### 2.3. The Fragment-Generation Algorithm

**Figure 1:**
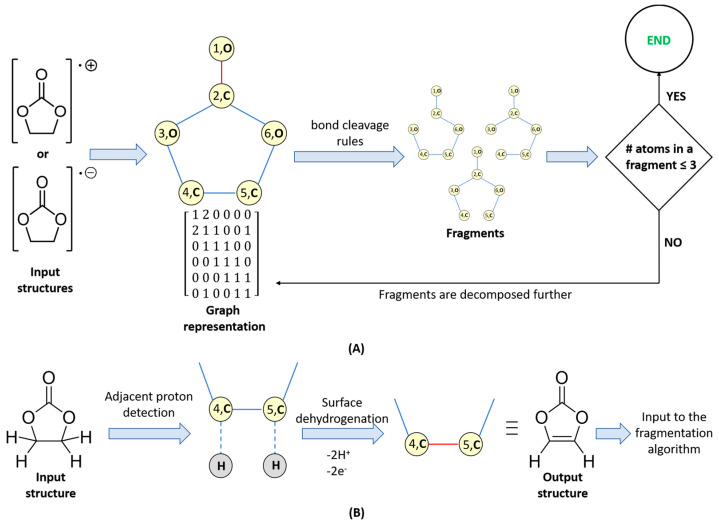
Schematic representation of the fragmentation (**A**) and surface dehydrogenation (**B**) steps.

**Figure 2:**
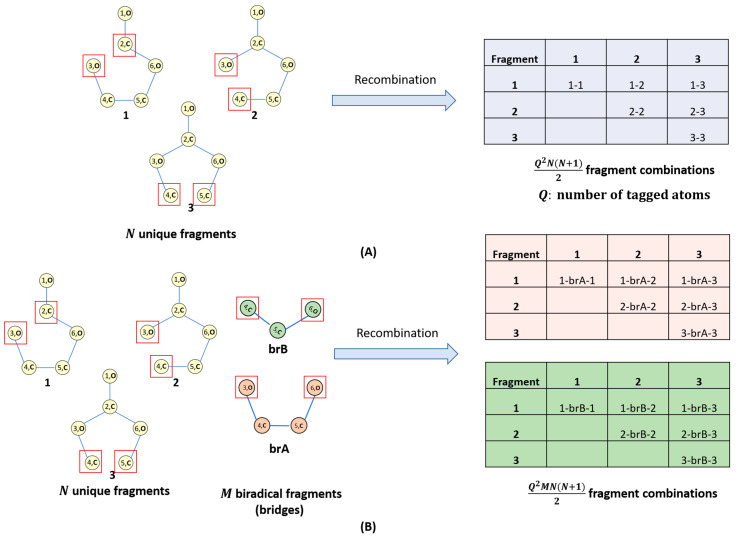
Schematic representation of the algorithm of recombination of two degradation products directly (**A**) or via a bridge (**B**). The atoms tagged during a fragmentation step are marked with red squares.

As mentioned in the introduction, one of the major reasons for battery performance loss is the electrolyte degradation; hence, the capability to foresee the degradation products is essential for the design of lithium- or sodium-ion cells with high cycling stability. For this purpose, we designed the graph theory-based algorithm illustrated in Figure 1. The algorithm takes into account the following transformations that are known to occur during battery charge–discharge cycling [10]:

(i) *Fragmentation:* On anodes and cathodes anion-radicals and cation-radicals are formed, respectively. The polar bonds (e.g., C–O) in these species are known to undergo cleavage, so these types of fragmentations are considered (Figure 1A). In the present work, C–C bond cleavages are avoided since C–C bonds are essentially non-polar and, thus, are generally robust. Yet, the algorithm implementation is versatile enough to enable different user-defined bond cleavage rules.
(ii) *Surface dehydrogenation:* Dehydrogenation of the electrolyte solvent may occur at inorganic electrode surfaces leading to the formation of unsaturated products (Figure 1B). For this step, an implicit surface presence is considered. The products generated are then fed to the fragmentation algorithm described in (i).
(iii) *Recombination and radical quenching:* Radical species can recombine, forming different oligomer products (Figure 2A,B). Radicals can also be quenched by the presence of a trace amount of water in the electrolyte solvent. This can also be taken into account.

The current implementation extensively uses the NetworkX library version 3.3 [32] for the storage and manipulation of graphs. The conversion of molecular structures (represented in MDL Mol file format) to pseudographs and vice versa are essential steps in the algorithm. The input MDL Mol files are read using the RDKit library v 2024.03.6 [33] and converted to a pseudograph where each atom in the input molecule is represented as a node (the number of hydrogen atoms connected to each atom is explicitly recorded), and the chemical bonds are represented as edges. The pseudograph adjacency matrix is constructed taking into account the bond order, e.g., for a double bond (*V_i_*, *V_j_*) *a_ij_* = 2 (see the adjacency matrix in Figure 1A). The total number of valence electrons (VE) corresponding to each structure (whether it is an input structure, a fragment, a surface dehydrogenation, or a recombination product) is computed as follows: $VE=\sum_{i}^{N}n_{i}v_{i}$, where *N* is the number of different atom types in the structure, *n_i_* is the number of *i*-th type atoms, and *v_i_* is the number of valence electrons of the *i*-th type atoms.

**Figure 3:**
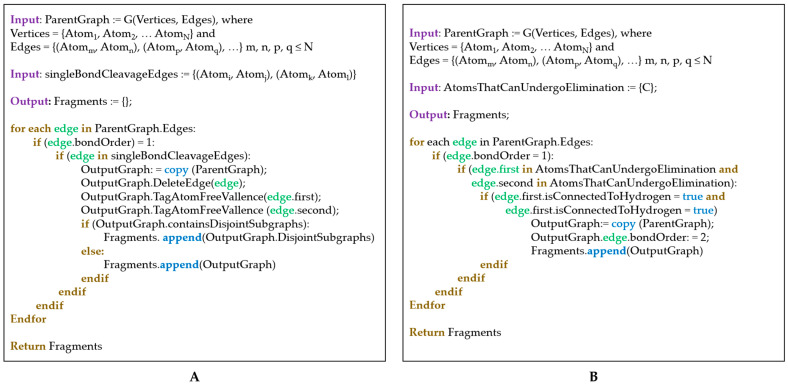
Pseudocode of the fragmentation (**A**) (see Figure 1A) and surface dehydrogenation (**B**) (see Figure 1B) algorithms.

The input to the fragmentation algorithm is either a cation-radical formed by cathodic oxidation of an electrolyte molecule (the latter normally being a neutral structure with an even number of valence electrons) or an anion-radical formed by anodic reduction in the same neutral electrolyte. For an electrolyte with *X* valence electrons (*X* is even), the cation-radical has (*X* − 1) valence electrons, while the anion-radical has (*X* + 1) valence electrons. For instance, a compound with molecular formula C_3_H_4_O_3_ has 3 × 4 (from the C-atoms) + 4 × 1 (from the H-atoms) + 3 × 6 (from the O-atoms) = 34 valence electrons, while [C_3_H_4_O_3_]^•+^ has 33 valence electrons and VE([C_3_H_4_O_3_]^•−^) = 35. The fragmentation (Figure 1A) and surface dehydrogenation (Figure 1B) steps are implemented by iterating through all graph edges (in the current pseudograph representation a graph edge is denoted as an unordered pair of atoms and a natural number that accounts for the bond order) and subjecting those edges to a pre-defined set of rules determining whether the chemical bond considered is able to undergo fragmentation or surface dehydrogenation. When a fragmentation is carried out, the atoms forming the cleaved bond are tagged. The tagged atoms are used in the recombination step (Figure 2) for binding different fragments. The pseudocodes of the fragmentation and surface dehydrogenation steps are shown in Figure 3. After each fragmentation step, the fragments graph representations are converted back to molecules (recorded as MDL MOL files). For this purpose:

(i) Atom types and connectivity information is extracted from the pseudograph and its adjacency matrix.
(ii) Charge and multiplicity of the fragments, are estimated as follows: if a fragmentation step leads to the generation of two separate fragments (1) the number of valence electrons of both fragments is calculated; (2) the sum of valence electron numbers of both fragments is calculated and compared to the number of valence electrons in the parent structure; and (3) if needed, the valence electron numbers of the fragments are adjusted so that VE(first fragment) + VE(second fragment) = VE(total). The adjusted valence electron numbers of the fragments are used to assign charge and multiplicity to each fragment. Consider, for example, the cleavage of the cation-radical **EC-2** (VE = 33, depicted in Figure S1, pathway III-o), leading to the formation of fragments **5** (CO_2_, VE_5_ = 4 + 2 × 6 = 16) and **8** (C_2_H_4_O, VE_8_ = 2 × 4 + 4 × 1 + 1 × 6 = 18). The sum of valence electrons of fragments **5** and **8** is VE_5_ + VE_8_ = 34, which is with one valence electron more than the number of valence electrons in **EC-2** and, hence, the VE—numbers of the fragments—needs to be adjusted with 1. As a result, the following pairs of fragments are possible: (1) [CO_2_]^•+^ (fragment **7**, VE_7_ = 15) and C_2_H_4_O (fragment **8**, VE_8_ = 18) so that VE_7_ + VE_8_ = 33; (2) CO_2_ (fragment **5**, VE_5_ = 16) and [C_2_H_4_O]^•+^ (fragment **6**, VE_8_ = 17) so that VE_6_ + VE_7_ = 33.

The fragmentation algorithm is run recursively on radicals and ion-radicals until the number of heavy atoms (i.e., atoms different from hydrogen) in the fragment becomes equal to 3. Closed-shell structures are not degraded further. Fragmentations leading to the formation of single atoms or methyl radicals/ion-radicals are not considered. All structures produced by the surface dehydrogenation (SD) step (Figure 1B) are subject to fragmentation. It should be noted that in the present work, the surface dehydrogenation is constrained to output a product only if the input structure contains at least one pair of adjacent C-atoms, both of which are connected to at least one hydrogen atom.

The recombination algorithm can be applied either to all radicals generated during the SD and fragmentation steps or to a subset of hand-picked radical fragments. The following recombination modes are considered:

(i) Recombination of N radical fragments with Q-tagged atoms leading to the formation of maximum Q^2^N(N + 1)/2 recombination products (Figure 2A);
(ii) Recombination of N radical fragments with Q-tagged atoms and M biradical bridges leading to the formation of up to Q^2^NM(N + 1)/2 recombination products (Figure 2B);
(iii) Recombination resulting in organic peroxide bonds is currently excluded;
(iv) Recombined centers are automatically untagged. Tagged atoms remaining after recombination are ‘quenched’ with hydrogen.

It should be noted that due to symmetry, the fragmentation steps may produce isomorphic structures. It is also common that similar compounds are generated along different fragmentation pathways. To address this issue, the IUPAC International Chemical Identifiers (InChI) [34,35] were used to pinpoint and discard fragments occurring more than once in the final set of degradation product candidates.

The degradation product candidates were exported as MDL Mol files with the RDKit library, and subsequently their geometry was pre-optimized with the AM1 [36] semi-empirical method, as implemented in HyperChem v7 [37].

### 2.4. Enthalpy Estimation

The algorithm described above generates many chemically viable parasitic products; however, in reality not all of them are formed. To determine which products are more probable, the reaction enthalpies of all fragmentation or recombination steps generated by the algorithm illustrated in Figure 1 were calculated using Hess’s law.

The enthalpies of formation of the possible parasitic products were computed by performing geometry optimization and frequency analysis using the hybrid density functional theory functional PBE0 (known in Gaussian as PBE1PBE) with 25% Hartree–Fock exchange [38] with Grimme’s D3BJ empirical dispersion scheme [39] and the 6-311++G(d,p) basis set. The unrestricted version of the DFT functional was used throughout. All DFT and semi-empirical calculations were performed in a vacuum. For all biradicals formed, both singlet and triplet ground states were optimized. The enthalpy of formation of each structure was estimated as follows:

$$H=E_{0}+ZPE+H_{trans}+H_{rot}+H_{vib}+RT,$$

where *E*_0_ is the total electronic energy, ZPE is the unscaled zero-point energy, and *H_trans_*, *H_ro_*_t_, and *H_vi_*_b_ are, correspondingly, the translational, rotational, and vibrational shares in the enthalpy. RT represents the work term converting the internal energy into enthalpy (T = 298 K). This computational protocol was chosen since it has been successfully applied to thermodynamic properties estimation of similar compounds [40]. All quantum chemical calculations were performed with the Gaussian 16 software package [41].

The electron affinity (EA) (enthalpy of the transformation from neutral structure to an anion-radical), the ionization potential (IP) (enthalpy of the transformation from neutral structure to a cation-radical), the bond dissociation enthalpy (BDE), and the surface dehydrogenation enthalpy (SDE) were computed using Hess’s low as follows:

EA = [H(cation-radical) + (H(e^−^)] − H (neutral structure) (1)

IP = H(anion-radical) − [H(e^−^) + H (neutral structure)] (2)

(3) $$\mathrm{BDE}=[\sum_{i=1}^{N}H(i-th\;fragment)]-H\;(\mathrm{parent}\;\mathrm{structure})$$

SDE = [H(unsaturated structure) + 2H(H^+^)) + 2H(e^−^)] − H(parent structure) (4)

The following values of enthalpy of electrons and protons in a vacuum were used: H(H^+^)) = 1.481 kcal/mol and (H(e^−^)) = 0.751 kcal/mol [42].

## 3. Results and Discussion

The algorithm was tested on two well-known cyclic and acyclic electrolyte solvents commonly utilized in metal-ion cells: ethylene carbonate and diglyme. Both oxidation, leading to the formation of cation-radicals, and reduction, resulting in the formation of anion-radicals, were considered. The ion-radicals were subject to fragmentation taking into account only C–O bond cleavages, since C–C bonds are stronger than C–O single bonds and, hence, harder to break. The ion radicals of surface dehydrogenation products of the solvents examined were also fed to the fragmentation algorithm. To assess the thermodynamic feasibility of the reduction, oxidation, fragmentation, and SD steps, the corresponding electron affinities, ionization potentials, bond dissociation enthalpies, and surface dehydrogenation enthalpies were calculated using Equations (1)–(4). It should be noted that all quantum chemical calculations were performed in a vacuum and hence the numerical values of SDE are overestimated because the computational scheme deployed disregards the stabilizing surface phenomena.

**Figure 4:**
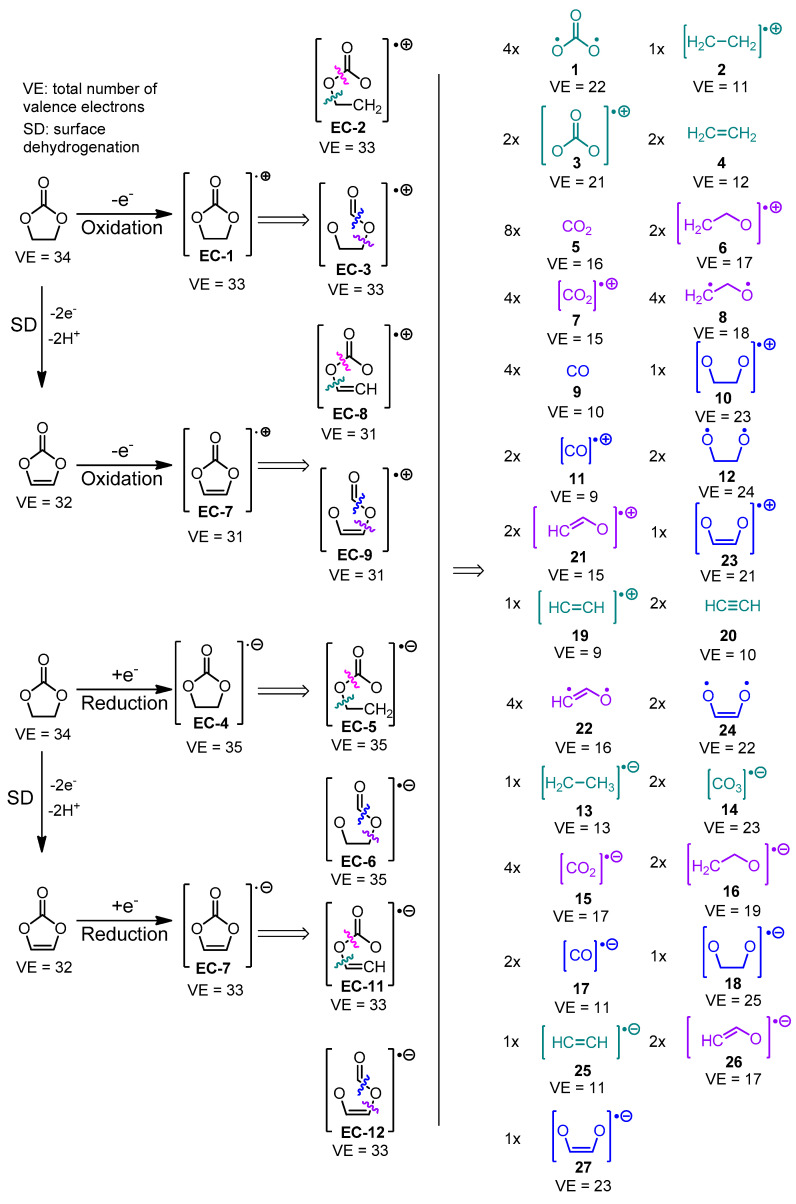
Fragmentation and surface dehydrogenation schemes (**left panel**) and products (**right panel**) of ethylene carbonate. Full fragmentation patterns and bond dissociation enthalpies are illustrated in Figures S1 and S2 in the Supporting Information. The number of different paths *N* leading to a given fragment is denoted as *N*x in front of the fragment structure.

**Figure 5:**
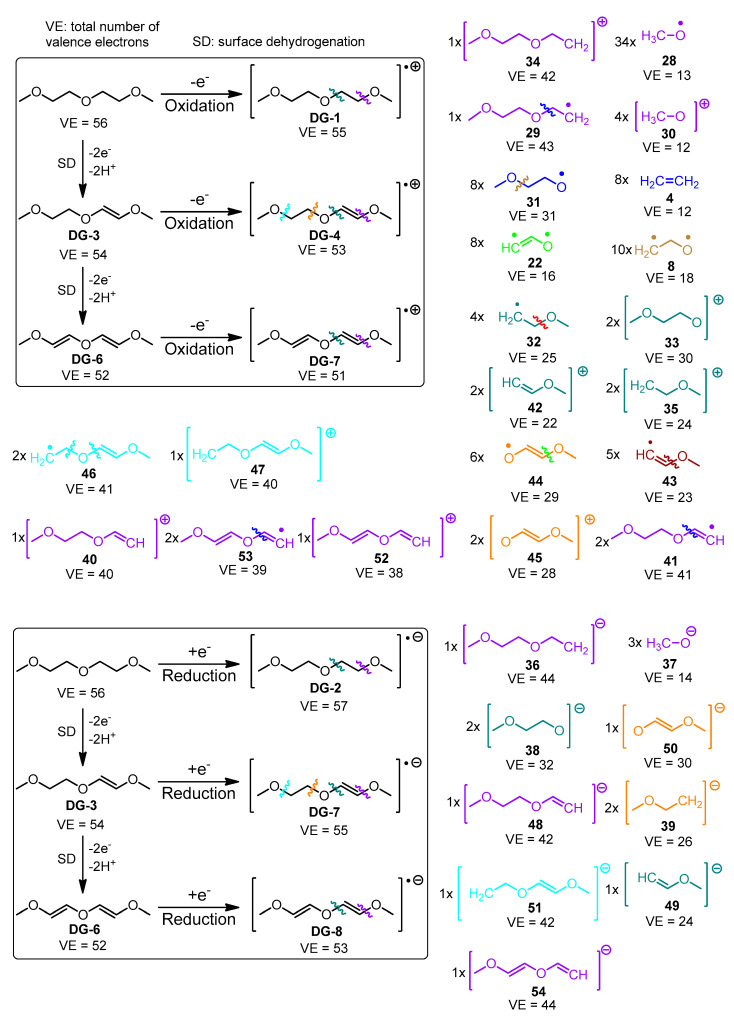
Fragmentation and surface dehydrogenation schemes (**boxed**) and products of diglyme. Full fragmentation patterns and bond dissociation enthalpies are present in Figures S3–S6 in the Supporting Information. The number of different paths *N* leading to a given fragment is denoted as *N*x in front of the fragment structure. The fragmentation products are outside the boxes.

The species derived from the fragmentation and the surface dehydrogenation of EC and DG are shown in Figure 4 and Figure 5. A step-by-step illustration of the degradation patterns of both solvents and the corresponding enthalpies associated with each step are presented in Figures S1 and S2 for EC and in Figures S3–S6 for DG.

The algorithm implemented in the present study generates all chemically plausible parasitic product candidates (i.e., structures obeying the valence rules). It can be expected that some of those species are far more likely to be detected in a given model system than others. The probability of detecting a given parasitic product candidate was assessed as follows:

First, the number of fragmentation pathways leading to a given parasitic product candidate were investigated—for instance, CO_2_ (**5**) is formed by eight different pathways during SD and fragmentation of ethylene carbonate (Figure 4, Figures S1 and S2). Different well-known gaseous side products such as ethene (**4**), ethyne (**20**), and carbon monoxide (**9**), along with the previously mentioned carbon dioxide, are generated by multiple pathways. Other parasitic species, commonly detected in ethylene and propylene carbonate-based lithium cells, such as [CO_2_]^•−^, are generated by four different pathways. Ilya A. Shkrob et al. have detected [CO_2_]^•−^, using electron paramagnetic resonance (EPR) in ethylene carbonate reduced by irradiation with 2.5 MeV electron beam to 3 kGy for 1 min at 77 K [43]. It should also be noted that different biradicals like **1** and **8** may also be generated via several degradation pathways.

Compared to EC, DG fragmentation produces a larger number of radical and ionic fragments of different size (Figure 5). The most common species in DG degradation are the methoxy radicals **28** that can be generated as a result of 34 different fragmentation steps, followed by fragment number **8** generated by 10 different fragmentation patterns. During DG surface dehydrogenation and fragmentation, no carbon dioxide is generated. The number of gaseous product candidates formed upon diglyme degradation is significantly smaller than the number of gaseous species formed by ethylene carbonate fragmentation. This aligns with the experimental data published by K. Westman et al. [44], who studied the gaseous products emitted by storing sodium under 1 M NaPF_6_ in diglyme in an isochoric container by continuously measuring the pressure change invoked by gas evolution. The equilibrium pressure measured corresponds (assuming ideal gas) to ca. 21 µmol per cm^2^ of Na, which is approximately two orders of magnitude smaller than the same quantity that may reach up to ~0.3 mmol/cm^2^ for sodium in 1 M NaPF_6_ in EC_50_:DMC_50_ [45].

Utilizing the results of the geometry optimization and the values of the computed enthalpies, the subsequent information is obtained: (a) in many cases, the geometry optimization of the most thermodynamically unstable and, hence, improbable products cannot be completed or leads to a dissipation of the structure being optimized; (b) the computed values of IP, EA, BDE, and SDE can be used to predict the most advantageous (thermodynamically) degradation pathway.

The following results of the geometry optimization need to be pointed out:

Structure **8** (a biradical) can only be successfully optimized as a singlet and a 3-member ring is formed in the optimized structure. Cyclic ethers have been observed in lithium cells using ethylene carbonate as an electrolyte solvent. G. Gachot et al. experimentally proved the formation of ethylene oxide upon reductive fragmentation by performing differential scanning calorimetry (DSC) combined with gas chromatography/mass spectroscopy (GC/MS) on 1 M LiPF_6_ in EC_50_:DMC_50_ electrolyte of a model half-cell using lithium foil as an anode and the commercially available SFG6 graphite (90 wt. %)/Super P carbon black (10 wt. %) as a cathode [46,47].

Carbon dioxide, carbon monoxide, and ethene are among the most energy-affordable products of ethylene and vinylene (derived by SD of EC) carbonate fragmentation in both oxidative and reductive fragmentation modes (Figure 4). These gases have been experimentally detected in different systems: (a) G. Gachot et al. [46] report the formation of these gases in Li/1M LiPF_6_ in EC_50_:DMC_50_/ SFG6 (90 wt. %) and Super P carbon black (10 wt. %) detected with DSC combined with GC/MS as described above. (b) Zhao et al. [48] utilized in situ differential mass spectroscopy techniques to detect ethylene carbonate gaseous degradation products in NCM622-Li, NCM811-Li, and NCM111-Li cells during cycling and resting. It was found that CO is evolved during oxidation, which is predicted as the most energetically affordable gaseous product of vinylene carbonate (VC) oxidative fragmentation. CO_2_ was also registered. (c) Roland Jung et al. perform online mass spectroscopy experiments (OLMS) on Li/1.5 M LiPF_6_ in EC/NMC622 and graphite/1.5 M LiPF_6_ in EC/NMC622 (it has to be noted that the electrolyte is liquid at room temperature due to the EC melting-point depression caused by LiPF_6_) to show that ethene is formed by reductive decomposition of EC during the SEI formation [49]. It has to be underscored that the most thermodynamically favorable reductive fragmentation pathway leads to the formation of ethene. According to the same literature source, CO_2_ and CO are accumulated during charging due to electrolyte oxidation.

Structure **22** (an unsaturated biradical) can be optimized only as a triplet, i.e., the formation of unsaturated 3-membered cyclic ethers is not probable.

Some cation-radicals (such as **10**) and cations (such as **33**, **40**, **42**, and **52**) are so unstable that the geometry optimization could not converge.

A hydride ion transfer leading to cation stabilization is observed during geometry optimization of some structures such as **30** and **33**.

The geometry of anion **51** cannot be optimized due to bond cleavage leading to the elimination of ethene.

The energy cost of oxidation is much higher than that of reduction for both EC and DG: the ionization potentials of EC and DG are 243.0 kcal/mol and 199.5 kcal/mol, respectively, while the corresponding electron affinities of the solvents are 7.0 kcal/mol and 12.3 kcal/mol. The same rule (i.e., reduction occurring more easily than oxidation) holds for the SD products of both solvents.

In both the oxidative and reductive degradation pathways, the cleavage of the C4-O3 bond in ethylene carbonate leading to the formation of products **EC-2** and **EC-5**, respectively, is thermodynamically more favorable than C2-O1 fragmentation, resulting in the generation of **EC-3** and **EC-6**, respectively. The fragmentation of vinylene carbonate (produced by surface dehydrogenation of EC), on the other hand, proceeds in the reverse manner: the cleavage of the C2-O1 bond leading to **EC-10** (oxidative fragmentation) and **EC-13** (reductive fragmentation) is energetically more advantageous than the C4-O3 bond cleavage, resulting in the formation of **EC-9** and **EC12**, respectively.

Westman et al. [44] concluded that the main degradation products of diglyme are alkoxy anions and polymerization products, since they were unable to detect uncharged soluble products with low molecular weight. The most energetically affordable reduction pathways lead to the generation of anionic species. Westman et al. also proposed a mechanism suggesting the formation of fragments similar to the ones generated by the algorithm discussed in the present study (Figure 5).

In oxidative degradation of DG and its surface dehydrogenated derivatives (the steps leading to the formation of a cation and a radical), it is more beneficial when the radical is localized at an oxygen center, while the positive charge resides at a carbon moiety. During reductive fragmentation, on the other hand, the bonds of the central oxygen atom are easier to dissociate.

The correspondence between algorithm-generated and experimentally detected EC degradation products is given in Table 1.

**Figure 6:**
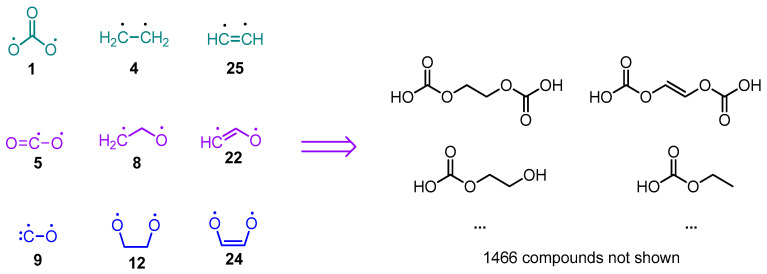
Recombination and radical quenching of EC-derived fragments. The lithium salts of the illustrated carboxylic acids have been found to be EC degradation products. The atoms tagged by the fragmentation algorithm are marked with dots.

The recombination algorithm (Figure 2) was benchmarked by generating all possible recombination products of the radical species yielded by the fragmentation of EC and VC. The recombination step produces a large number of electrolyte degradation side product candidates, some of which are shown in Figure 6.

Lithium salts of the carboxylic acids depicted in Figure 6 have been found in the electrolyte of silicon lithium-ion batteries and studied using nuclear magnetic resonance [50].

**Figure 7:**
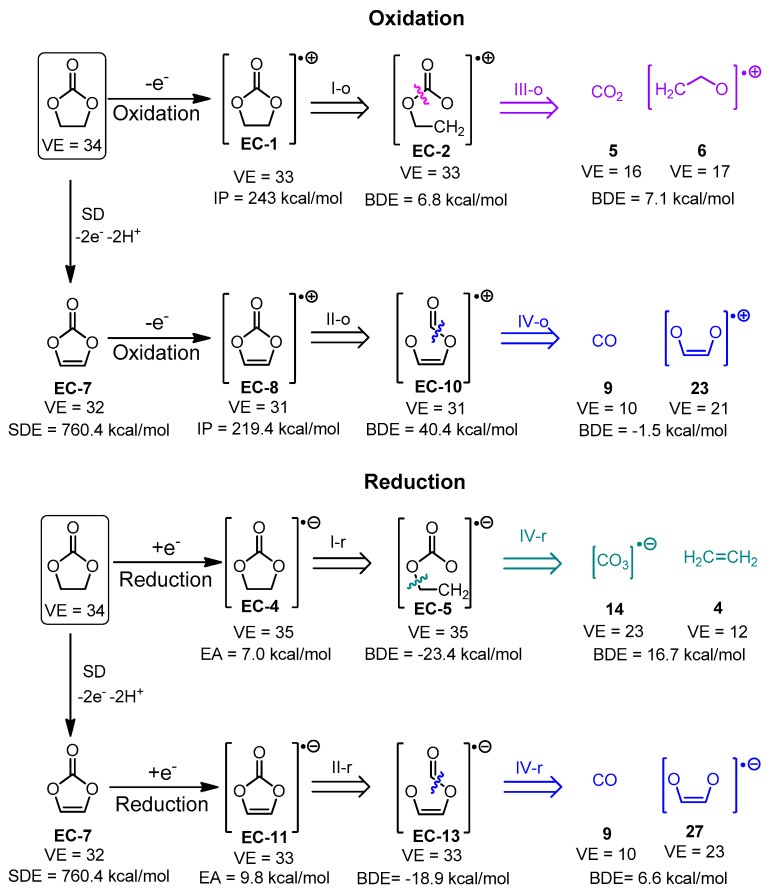
The most thermodynamically feasible fragmentation pathways of ethylene carbonate.

**Figure 8:**
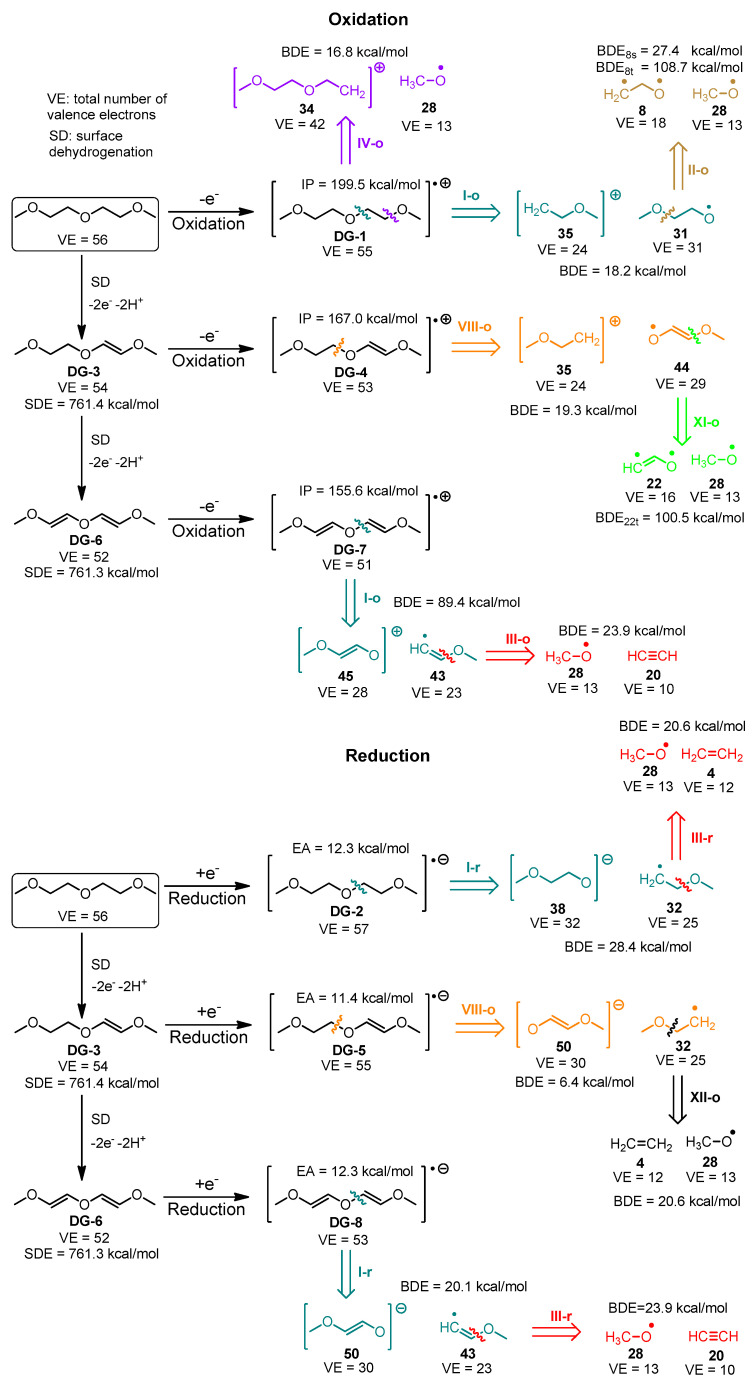
The most thermodynamically feasible fragmentation pathways of diglyme.

The most thermodynamically favorable pathways for EC and DG fragmentation are shown in Figure 7 and Figure 8, respectively.

## 4. Conclusions

This study presents an algorithm that allows the rapid deduction of the structures of possible electrolyte solvent degradation products using a set of user-defined bond cleavage rules. The generation time of all fragments and recombined products of electrolyte solvents is in the order of seconds on a widely accessible hardware. The proposed algorithm is tested by predicting the degradation products of two frequently utilized electrolyte solvents: EC and DG. We judge the abundance of particular products by the number of routes leading to them and the corresponding reaction enthalpies estimated with DFT. The experimental conditions cannot be included in the graph-theoretical approach, but we validate the approach by comparison with available experimental data, which confirm our forecasts.

The most energetically favorable oxidation and reduction pathways are summarized in Figure 7 and Figure 8. Upon oxidation, the energetically preferred products are carbon dioxide and cation radicals (Figure 7). The reduction in ethylene carbonate yields a carbonate anion-radical and ethene. The availability of the electrode surface opens an additional corridor for degradation with the formation of carbon monoxide under oxidation and reduction. Compared to EC, the DG degradation results in the formation of methoxy radicals and O-containing organic products with less gaseous products evolved (Figure 8). The same result is observed even after the presence of the electrode surface is implicitly taken into account. The predicted degradation products are in good agreement (Table 1) with the experimentally detected products, confirming the validity of the proposed algorithm.

The novelty of the algorithm is not in the devising of new methods but in combining unemployed approaches for degradation products prediction, such as the graph-theory methodology and the valence electrons splitting schemes. Valuable assets are also the code simplicity and adaptiveness, as well as the speed of performance.

In general, the algorithm, combined with quantum chemistry methods and the literature search for spectra of the fragments proposed, may simplify the interpretation of different in situ experiments. In perspective, the possible parasitic reactions of novel electrolytes can be forseen and appropriate additives for radical quenching may be proposed. Moreover, the algorithm can be used to predict the oxidation and reduction in novel electrolyte solvents for which no experimental data are available. Modeling of graph theory-derived degradation products of electrolytes with single and binary solvents for sodium-ion batteries are in progress.

## Figures and Tables

**Table 1:** Comparison between experimental and algorithm-generated degradation products.

Degradation Product	Algorithm Steps	Electrochemical Cell Configuration	Detection Experimental Conditions	Ref.
[CO_2_]^•−^	Figure S1/IV-o and IV-r Figure S2 IV-o and IV-r	-	EPR in ethylene carbonate reduced by irradiation with 2.5 MeV electron beam to 3 kGy for 1 min at 77 K	[43]
Ethylene oxide	Figure S1/III-r	Li/1 M LiPF6 in EC50:DMC50/ SFG6 (90 wt. %) and Super P carbon black (10 wt. %)	DSC of lithiated electrode material combined GC/MS study of the compounds corresponding to each DSC peak	[46,47]
CO and CO_2_	Figure S1/III-o, III-r, IV-o, IV-r, V-o, V-r; Figure S2/III-o, III-r, IV-o, IV-r, V-o, V-r	Li/1 M LiPF6 in EC50:DMC50/ SFG6 (90 wt. %) and Super P carbon black (10 wt. %)	DSC combined GC/MS study of the compounds corresponding to each DSC peak	[46]
CO and CO_2_	Figure S1/III-o, III-r, IV-o, IV-r, V-o, V-r; Figure S2/III-o, III-r, IV-o, IV-r, V-o, V-r	NCM622-Li, NCM811-Li and NCM111-Li cells	In situ differential mass spectroscopy techniques	[48]
Ethene	Figure S1/I-o and I-r	Li/1.5 M LiPF6 in EC/NMC622 and graphite/1.5 M LiPF6 in EC/NMC62	Online mass spectroscopy experiments (OLMS)	[49]
Total gas evolution	Figures S1 and S2—all paths leading to gaseous products	Na/in 1 M NaPF6 in EC50:DMC50 /hard C	Continuous measurements of the pressure change invoked by gas evolution	[45]

## Data Availability

The original contributions presented in the study are included in the article; further inquiries can be directed to the corresponding authors.

## Supplementary Materials

The following supporting information can be downloaded at: https://www.mdpi.com/article/10.3390/ma18040832/s1: Figure S1: Fragmentation pathways of ethylene carbonate; Figure S2: Fragmentation pathways of ethylene carbonate surface dehydrogenation product; Figure S3: Diglyme fragmentation pathways; Figure S4: Surface dehydrogenation of diglyme and oxidative fragmentation pathways of the first surface dehydrogenation product (DG3); Figure S5: Reductive fragmentation of DG-3; Figure S6: Fragmentation pathways of the DG-6 diglyme surface dehydrogenation product.
