# Supplementary material for: Graph-Theory Algorithm for Prediction of Electrolyte Degradation Reactions in Lithium- and Sodium-Ion Batteries
Source: Materials (Basel). 2025 Feb 14;18(4):832. doi: 10.3390/ma18040832 (PMC11857540; doi:10.3390/ma18040832)
Supplement: Supplementary file 1 [file materials-18-00832-s001.zip › materials-3465304-supplementary.pdf]

materials

Supporting information

Graph-Theory Algorithm for Prediction of Electrolyte Degradation Reactions in Lithium- and Sodium-Ion Batteries

Lyuben Borislavov [1,*], Alia Tadjer [1,2,*] and Radostina Stoyanova [1]

[1] Institute of General and Inorganic Chemistry, Bulgarian Academy of Sciences, 1113 Sofia, Bulgaria; radstoy@svr.igic.bas.bg
[2] Faculty of Chemistry and Pharmacy, University of Sofia, 1164 Sofia, Bulgaria
* Correspondence: lborislavov@svr.igic.bas.bg (L.B.); tadjer@chem.uni-sofia.bg (A.T.)

9

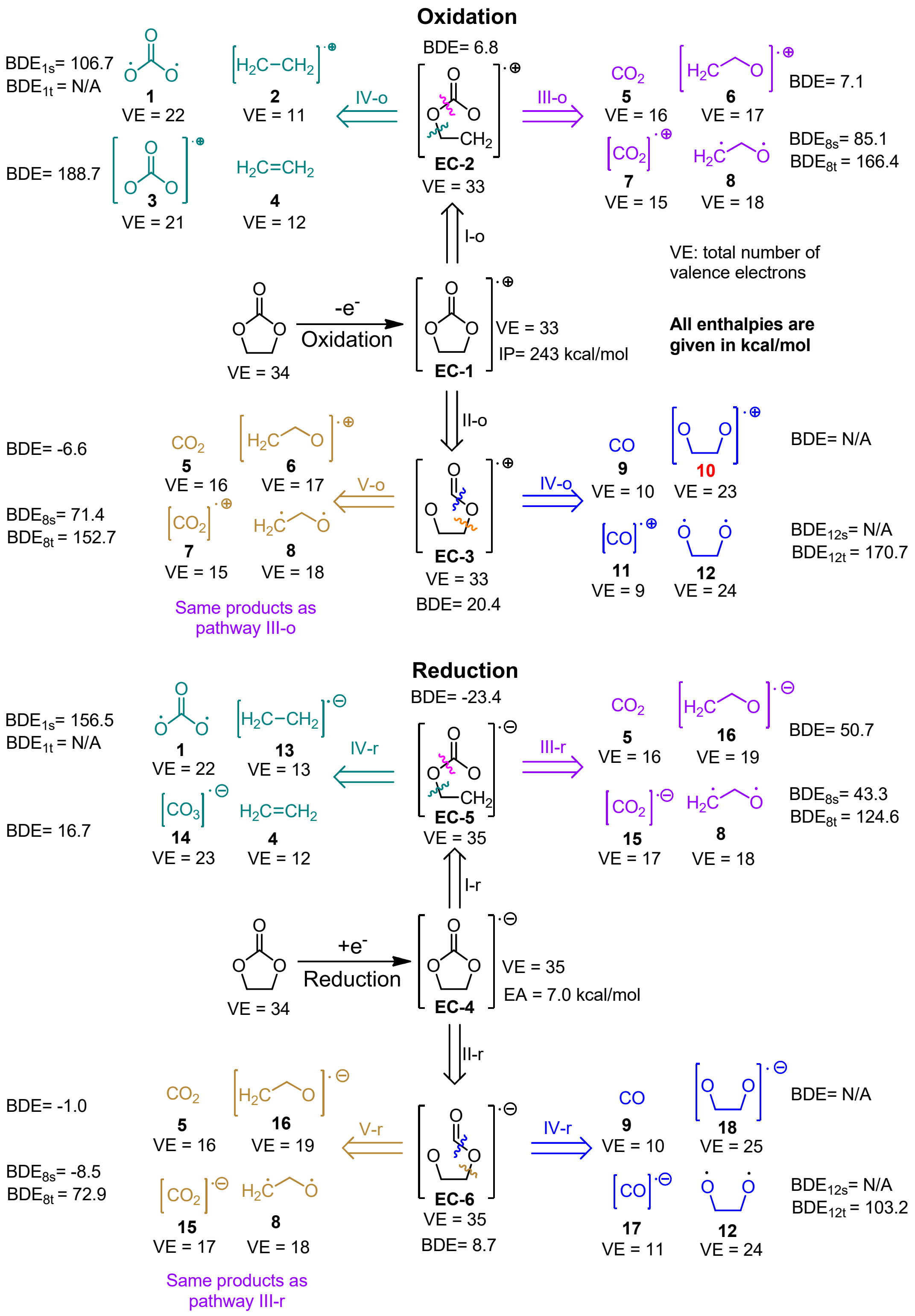

Figure S1. Fragmentation pathways of ethylene carbonate: ionization potential (IP), electron affinity (EA) and bond dissociation enthalpy (BDE) are given in kcal/mol. 10 11

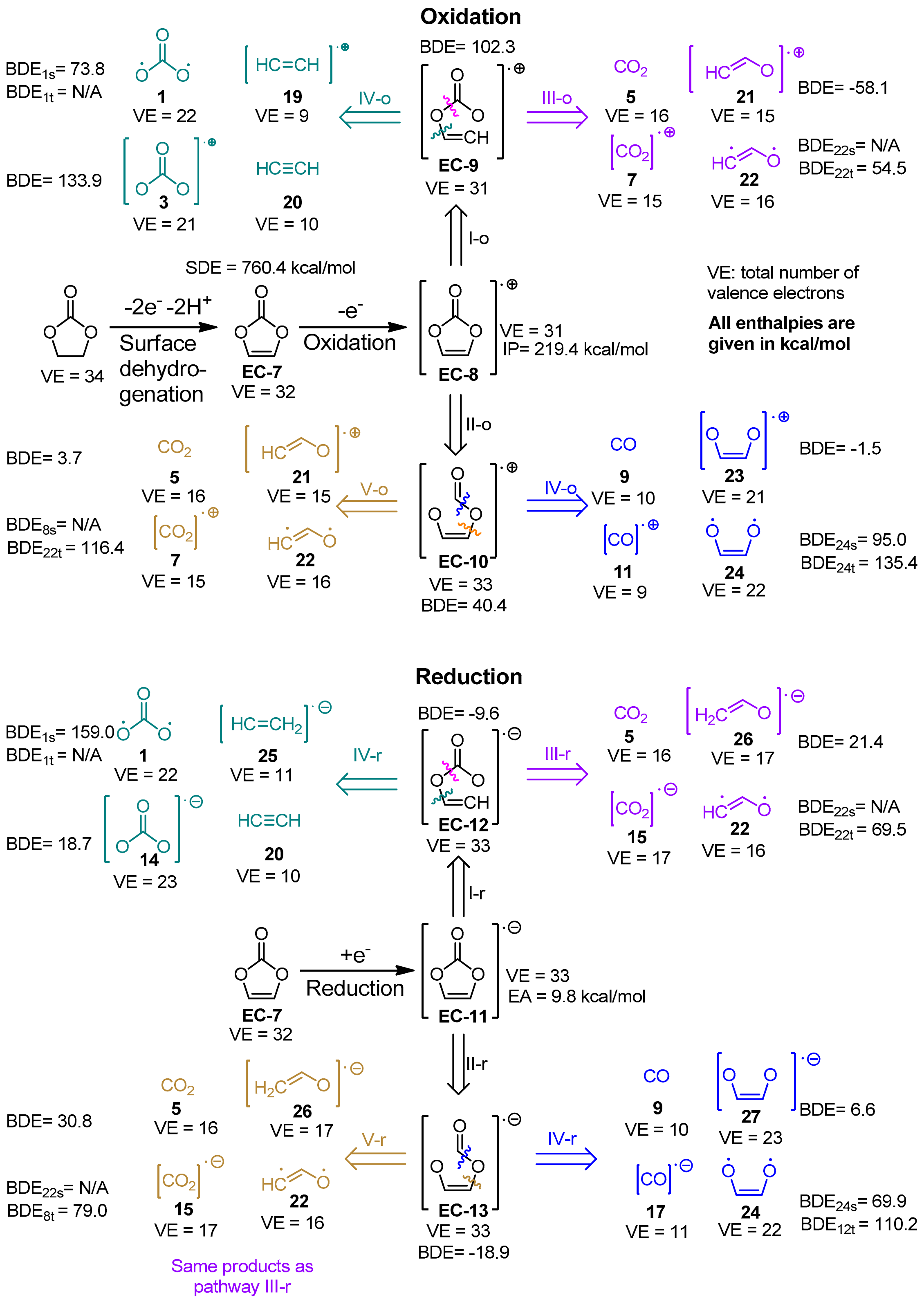

Figure S2. Fragmentation pathways of ethylene carbonate surface dehydrogenation product: ionization potential (IP), electron affinity (EA) and bond dissociation enthalpy (BDE) are given in kcal/mol.

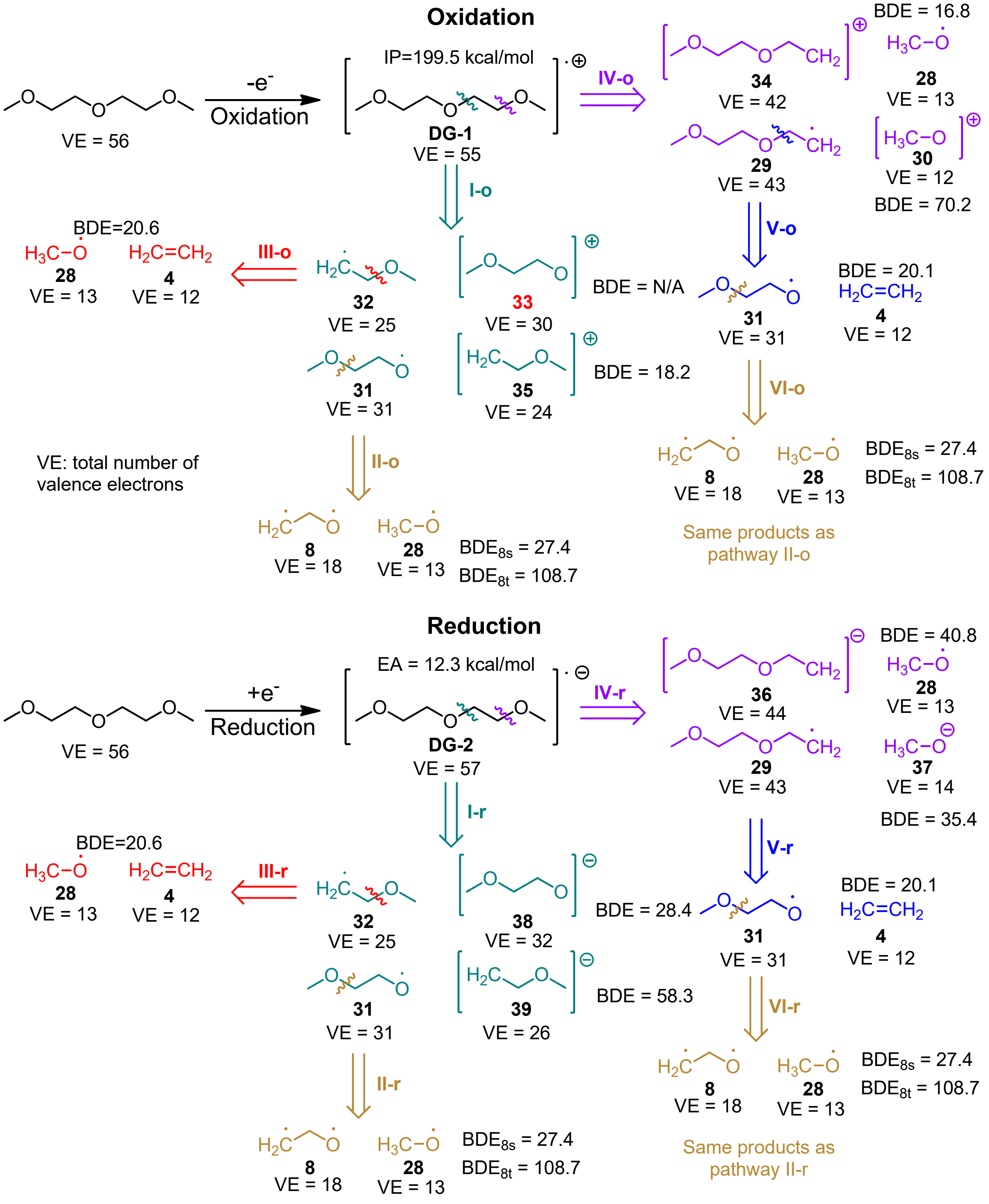

Figure S3. Diglyme fragmentation pathways: ionization potential (IP), electron affinity (EA) and bond dissociation enthalpy (BDE) are given in kcal/mol..

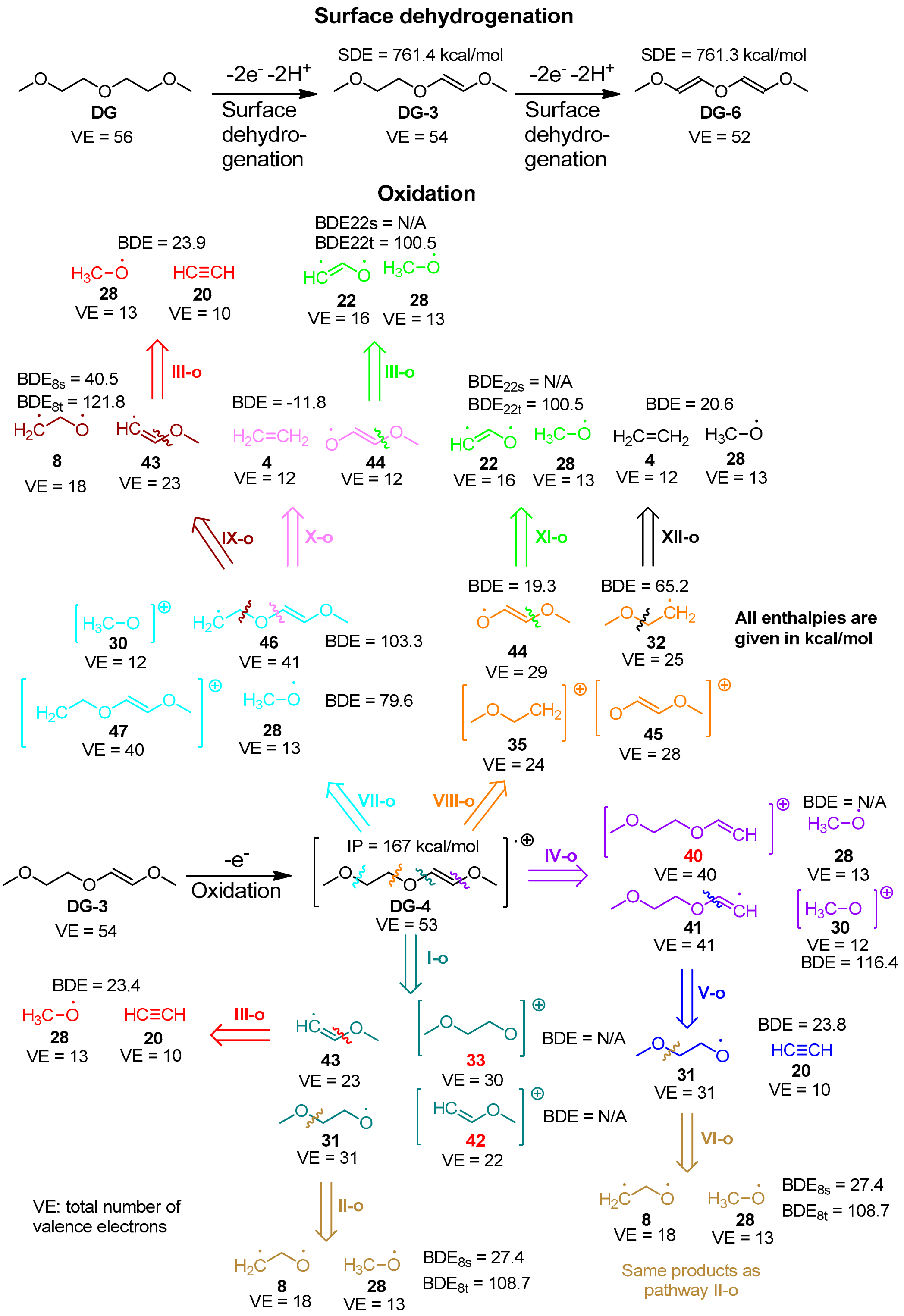

Figure S4. Surface dehydrogenation of diglyme and oxidative fragmentation pathways of the first surface dehydrogenation product (**DG3**). Ionization potential (IP), electron affinity (EA) and bond dissociation enthalpy (BDE) are given in kcal/mol..

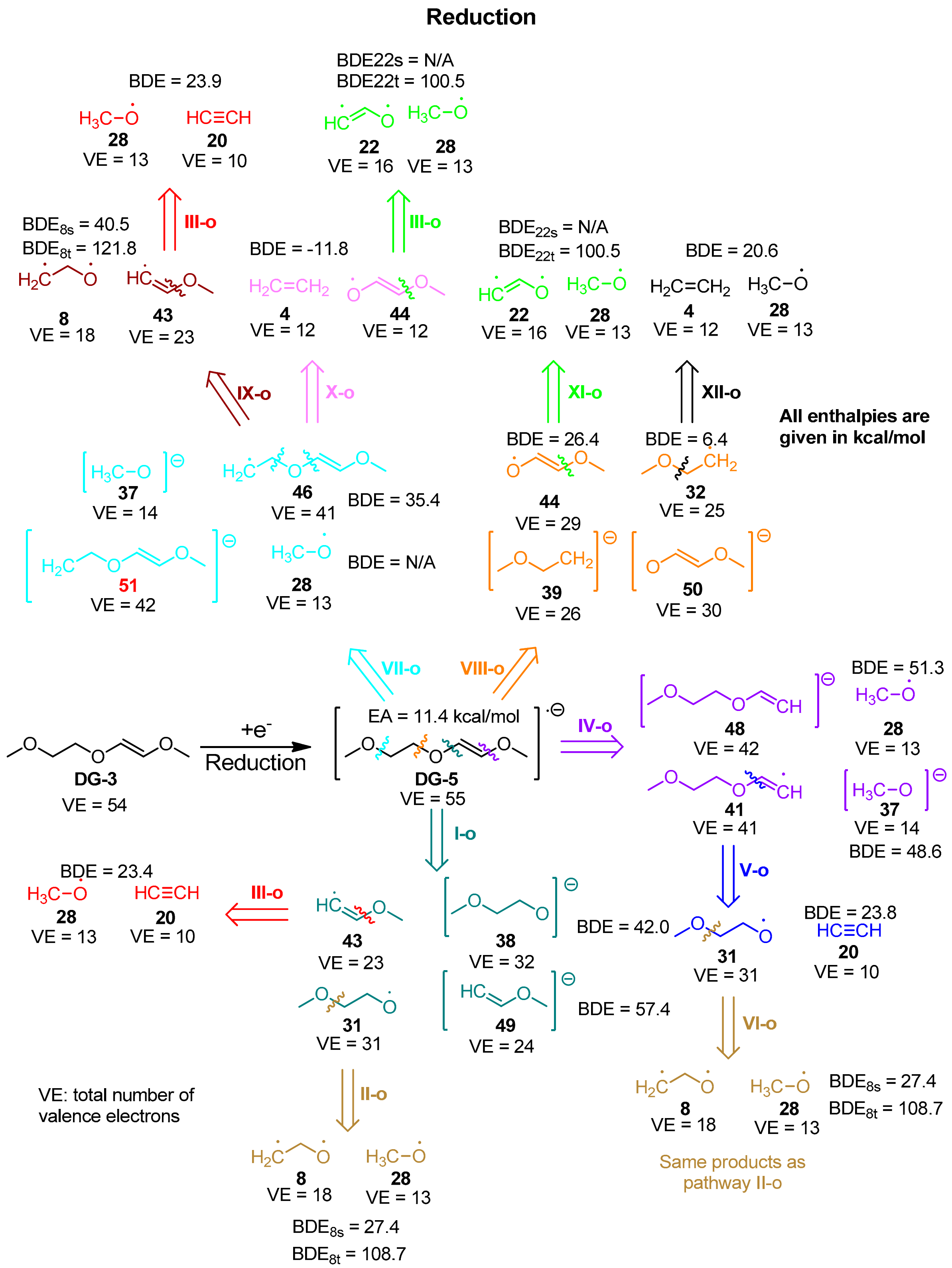

24

Figure S5. Reductive fragmentation of **DG-3**. Ionization potential (IP), electron affinity (EA) and bond dissociation enthalpy (BDE) are given in kcal/mol.. 25 26

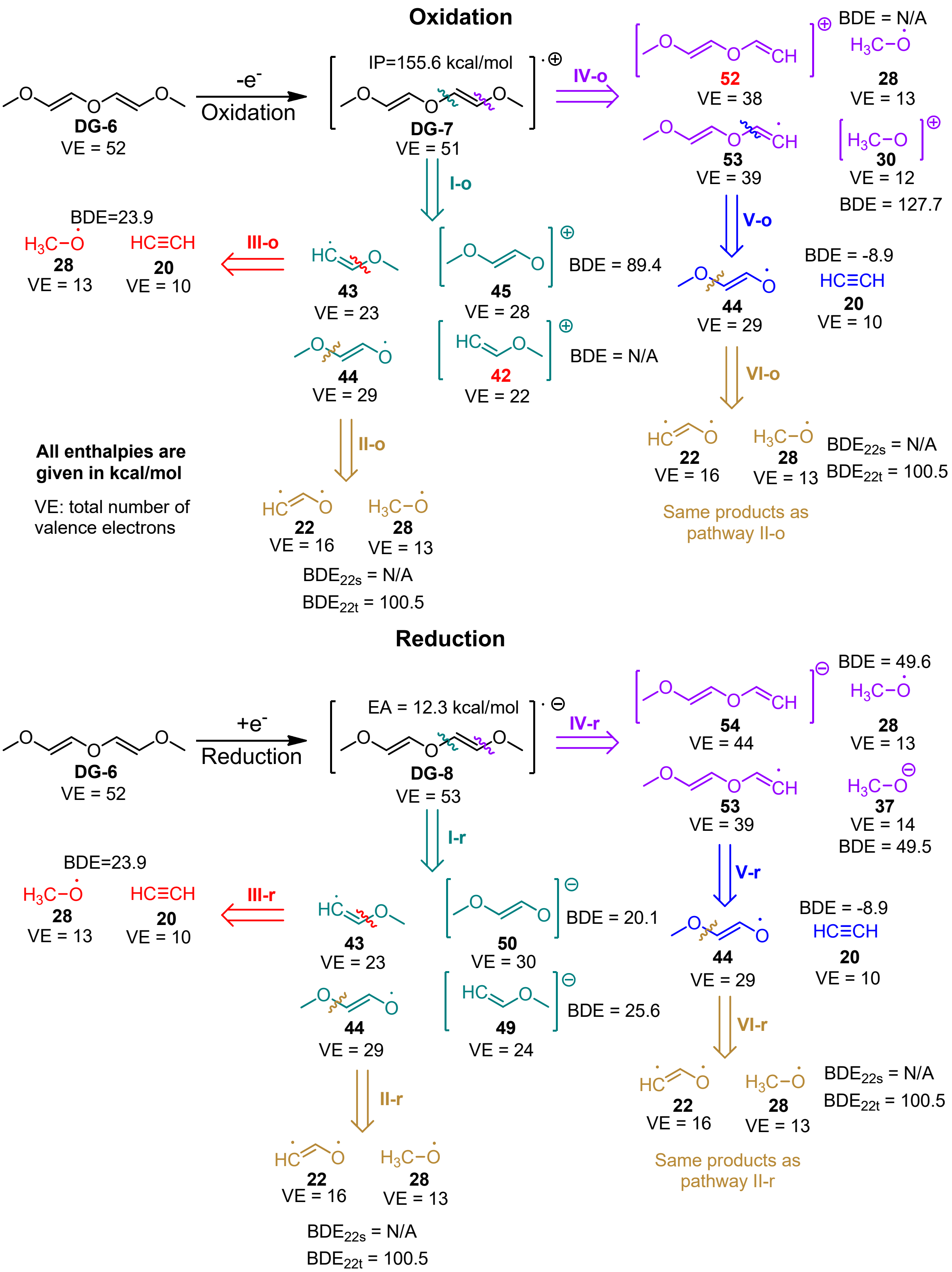

Figure S6. Fragmentation pathways of the **DG-6** diglyme surface dehydrogenation product. Ionization potential (IP), electron affinity (EA) and bond dissociation enthalpy (BDE) are given in kcal/mol..
